# HIV treatment cascade among female entertainment and sex workers in Cambodia: impact of amphetamine use and an HIV prevention program

**DOI:** 10.1186/s13722-017-0085-x

**Published:** 2017-09-05

**Authors:** Sokunny Muth, Aynar Len, Jennifer L. Evans, Maly Phou, Sophal Chhit, Yuthea Neak, Song Ngak, Ellen S. Stein, Adam W. Carrico, Lisa Maher, Kimberly Page

**Affiliations:** 1 FHI360, Phnom Penh, Cambodia; 2 Department of Epidemiology and Biostatistics, and Global Health, University of California, San Francisco, San Francisco, CA USA; 3 Department of Mental Health and Substance Abuse, Ministry of Health, Phnom Penh, Cambodia; 4 National Authority for Combating Drugs, Phnom Penh, Cambodia; 5 Public Health Sciences and Psychology, University of Miami, Coral Gables, FL USA; 6 The Kirby Institute for Infection and Immunity, UNSW Australia, Sydney, Australia; 7 Division of Epidemiology, Biostatistics and Preventive Medicine, Department of Internal Medicine, University of New Mexico, Albuquerque, NM USA

**Keywords:** HIV, Cambodia, Entertainment workers, Female sex workers, Amphetamines, HIV continuum of care, Treatment cascade

## Abstract

**Background:**

HIV prevalence remains high in Cambodia among female entertainment and sex workers (FESW), and amphetamine-type stimulant (ATS) use significantly increases risk of infection. A successful continuum of care (CoC) is key to effective clinical care and prevention. This study aimed to describe the HIV CoC in HIV-positive FESW. We examined CoC outcomes among HIV-positive FESW participating in the Cambodia Integrated HIV and Drug Prevention Implementation (CIPI) study, being implemented in ten provinces. CIPI is a trial aimed at reducing ATS use concomitant with the SMARTgirl HIV prevention program.

**Methods:**

From 2013 to 2016, 1198 FESW ≥ 18 years old who reported multiple sex partners and/or transactional sex were recruited. We identified 88 HIV-positive women at baseline. We described linkage to care as 12-month retention and viral suppression (<1000 copies/mL). Logistic regression analyses were conducted to examine correlates of retention in care at 12 months, and viral suppression.

**Results:**

Median age of the 88 HIV-positive women was 32 years [interquartile range (IQR) 28, 35]; 50% were working in entertainment venues and 50% as freelance sex workers; 70% reported SMARTgirl membership. In the past 3 months, women reported a median of 15 sex partners, 38% reported unprotected sex, and 55% reported using ATS. Overall, 88% were receiving HIV care, 83% were on antiretroviral therapy, 39% were retained in care at 12 months, and 23% were virally suppressed. SMARTgirl membership was independently associated with fourfold greater odds of 12-month retention in care (AOR = 4.16, 95% CI 1.38, 12.56). Those at high risk for an ATS use disorder had 91% lower odds of 12-month retention in care (AOR = 0.09, 95% CI 0.01, 0.72). Viral suppression was independently associated with SMARTgirl membership, older age, reporting of STI symptoms, worse symptoms of psychological distress, and greater numbers of sex partners.

**Conclusions:**

This is the first study to characterize the HIV CoC in Cambodian FESW. While most women were successfully linked to HIV care, retention and viral suppression were low. Tailored programs like SMARTgirl, targeting the broader population of HIV-positive FESW as well as interventions to reduce ATS use could optimize the clinical and population health benefits of HIV treatment.

*Trial registration* This work reports data collected as part of a trial: NCT01835574. This work does not present trial results

## Background

HIV risk remains high in Cambodia among FESW. High numbers of sexual partners and non-injection ATS use are associated with elevated HIV risk and infection rates in this population [1–3]. Cambodia has achieved substantive progress in reducing the spread of HIV at a population level; the estimated number of new infections has fallen 95% from a peak of 24,348 in 1995 to 651 in 2015 [4]. Comprehensive deployment of basic HIV prevention programs including condom promotion, HIV voluntary counseling and testing, and high coverage of HIV antiretroviral treatment (ART), have contributed to these declines [5]. However, women engaged in entertainment and sex work remain disproportionately impacted [3]. In addition to individual risks, structural factors present challenges for HIV prevention among FESW. Anti-trafficking legislation aimed at suppressing human trafficking and sexual exploitation amplified HIV risks: brothel closures and migration of working women to entertainment venues and street-based sex work had negative impacts on access to HIV and STI prevention and health services [6]. Stigma, discrimination and violence further contribute to risk and negative health outcomes [7–9].

Cambodia has a national strategic goal of achieving elimination of new HIV infections by 2020. The National Center for HIV/AIDS, Dermatology and STI (NCHADS) Strategic Plan for HIV/AIDS and STI Prevention and Control in the Health Sector 2015–2020 outlines core phased strategies based on enhancing the HIV CoC including prevention, linkage and retention in care, enhanced quality of care and elimination of perinatal transmission [4]. The HIV CoC has been widely used to document engagement in care among people living with HIV [10]. The CoC includes measurable indicators from HIV detection through linkage and engagement in care and treatment. While measurement can vary, the framework can be applied widely or to key populations to inform HIV responses and document health disparities as it identifies losses at each step along the continuum [11–15]. CoC data on key populations, especially in FESW, are limited [16, 17].

The multiple circumstances and exposures that contribute to high risk for HIV infection in Cambodian FESW may also impact success along the HIV CoC. In particular, the high prevalence of problematic ATS use represents a major barrier to HIV treatment as prevention in this population. This is supported in part by prior research conducted predominantly in the United States (U.S.) where persons with HIV who use stimulants such as methamphetamine experience profound difficulties along the HIV CoC. People with HIV infection who use stimulants initiate ART at lower CD4 counts [18], are less likely to remain engaged in HIV care [19], and report difficulties with ART adherence that contribute to substantially elevated HIV viral load [18]. They may also experience faster disease progression [20, 21]. A threefold greater rate of AIDS-related mortality has been observed in crack-cocaine using women [22]. Women with HIV infection who use stimulants in the U.S. are also more likely to report engaging in HIV transmission risk behavior [23, 24], resulting in increased odds of onward HIV transmission where viral load is greater than 200 copies/mL [25]. We examine CoC outcomes among a sample of HIV-positive FESW participating in the Cambodia Integrated HIV and Drug Prevention Implementation (CIPI) Study being implemented in ten provinces in Cambodia [26, 27].

### Study design and methods

#### Study sample and setting

In this paper, we restrict analyses to HIV-positive women who were enrolled in the CIPI Study. The CIPI Study targeted women at high risk of HIV infection in 10 provinces of Cambodia: female entertainment and sex workers (FESW). Specifics on the population and methods have been described in detail in a previous publication defining the protocol for the CIPI Study [26]. In brief, the study recruited and enrolled FESW who: (1) were biologically female; (2) aged ≥18 years; (3) reported ≥2 different sexual partners and/or transactional sex within the last month; (4) understood spoken Khmer language; and (5) were able to provide voluntary informed consent. Transactional sex was defined as exchanging sex for money, goods, or social favors. FESW work in a variety of venues including beer gardens, bars, massage, karaoke and other ‘entertainment’ establishments, as well as in other “freelance” venues including parks and guesthouses. In all provinces, CIPI outreach workers recruited women from multiple locations and locales where FESW were known to congregate or work. Outreach workers conducted a preliminary verbal screening for eligibility, described the study, answered questions, and invited women to attend the local study site (SMARTgirl Club) for eligibility evaluation and, if interested, study participation. HIV infection status and testing was not a criterion for participation in the CIPI study.

The CIPI Study was implemented within the SMARTgirl HIV prevention platform [26, 27], a widely disseminated ‘standard of care’ program launched nationally in 2008 to reduce HIV risk in women working in the high-risk entertainment sector [28]. SMARTgirl clubs exist in most provinces, and local community-based organizations maintain them and provide outreach to local women. The SMARTgirl program uses targeted communication tools and club events to disseminate health information, conduct and promote HIV testing, and referrals to health services including HIV care, sexually transmitted infection and reproductive health services. The CIPI Study integrated an ATS intervention and microenterprise opportunity within the existing SMARTgirl platform (described in [26, 27]).

#### Measures

At CIPI study visits, women were asked to complete an interviewer-administered survey regarding: socio-demographic information; work history including sex and entertainment work settings; sexual risk exposures, including number and types of partners, and condom use, and ATS exposures, both lifetime (at baseline) and in the past 3 months (at baseline and follow-up interviews); alcohol use, history of HIV testing and knowledge of antibody status; age and circumstances of first sex; experience of violence; health service utilization for HIV, STI and reproductive health; economic well-being; empowerment; and self-efficacy. ATS and alcohol use were assessed using the Alcohol Smoking and Substance Involvement Test (ASSIST), a validated screening measure for assessing alcohol and other substance use disorders [29]. Using validated cut off points for each substance use involvement score, the ASSIST indexes low, moderate, or high risk for the presence of disordered patterns of use. Psychological distress was assessed using the Kessler-10 (K-10), a validated, 10-item screening measure for mild, moderate, or severe distress that has shown superior validity in identifying depressive and anxiety disorders [30]. Each participant was instructed and asked to provide a urine specimen to be tested for ATS metabolites (i.e., methamphetamine and amphetamine). Urine is tested for adulteration using Specimen Validity Testing (SVT) (Innovation, Inc., San Diego, CA), and for ATS metabolites using the Innovacon Multi-Drug Screen Test Panel Dip Kit (Redwood Toxicology Laboratory, Inc., Santa Rosa, CA). Women were tested for prostate-specific antigen test (PSA), a biomarker of recent unprotected sex [31]. HIV testing was conducted at study entry (and subsequent visits) using a rapid antibody test in accordance with Cambodian National Guidelines, on women who consented to testing. Self-reported HIV testing history and results were obtained in interviews. We also linked all participants’ names (with consent) to national records at the National Center for HIV, STD and Dermatology (NCHADS) to validate self-reported results, obtain results on participants who declined testing, and obtain date of first HIV detection for all women in the CIPI study, Participants who elected to receive HIV testing received concomitant HIV risk reduction counseling, and participants testing positive results were referred to HIV care services. Since HIV testing was voluntary within the CIPI study, review with NCHADS allowed us to examine and confirm HIV testing history on all women in the study. Time since HIV diagnosis was determined from this record review or for women who received a new HIV-positive results in the CIPI study, we used the date of that test to calculate the time since HIV diagnosis.

Detailed information on HIV CoC outcomes, including date of ART uptake and viral load was obtained through linking CIPI participant identifiers with SMARTgirl records and national surveillance data from NCHADS. We constructed a 5-stage HIV CoC with the following events: (1) diagnosed with HIV; (2) enrolled in HIV care; (3) initiation of ART; (4) retention in care for 12 months after initiating ART; and (5) viral suppression (i.e., HIV viral load less than 1000 copies/mL) [10, 12, 13]. Enrolled in HIV care was defined as having ever seen a medical practitioner (Nurse/Medical Assistant/MD) for HIV. ART initiation was defined as having started ART under the care of a medical practitioner. Retention was defined as having attended an appointment with an HIV medical practitioner within 12 months of ART initiation. NCHADS defines viral suppression as having at least one viral load test result less than 1000 copies/mL consistent with WHO guidelines [10].

#### Analyses

Cascade staging was assessed using both a “single” denominator (the number of HIV-positive women in the cohort) estimating loss as a proportion of those diagnosed, and with a “running” denominator wherein each indicator is assessed as a percent loss from the previous stage. The cascade figure was prepared using an “HIV States and Transitions” framework to summarize ‘states’ of participants within each state [13]. For bivariate analyses, we used Chi square and the Wilcoxon rank-sum tests, to test associations between categorical and continuous variables of interest, respectively, and CoC outcomes. Multivariable logistic regression analyses were performed to assess independent associations with 12-month retention in care following ART initiation as a primary outcome. Bivariate and multivariable analyses used robust standard errors. We also examined correlates of viral suppression as an exploratory outcome. Adjusting for time since HIV diagnosis, recent STI symptoms, and age at first sexual intercourse, factors were entered into a multivariable regression models if significantly associated (*p* ≤ 0.10) in bivariate analyses, and retained if adjusted *p* values were ≤0.05. All statistical analyses were performed using Stata software, version 13.0 (StataCorp, College Station, TX).

#### Ethics

This study population experiences marginalization and is engaged in illegal behaviors. Sensitive behavioral and biological data were collected of this population. The study was conducted in compliance with ethical principles for human subjects research promoted by the Declaration of Helsinki developed by the World Medical Association [32]. Various procedures were adopted to ensure additional protection of all the women screened and enrolled in the trial including waiver of signed written informed consent. The consent process was conducted carefully to ensure potential participants understood the research objectives, procedures, risks and benefits, reimbursement, costs, and alternatives, and a short verbal assessment was conducted to ensure understanding. All women consented to have study information linked to NCHADS HIV records. The study protocol was reviewed and approved by the Cambodian National Ethics Committee, and the Institutional Review Boards (IRBs) of the University of California San Francisco and FHI360. The University New Mexico Health Sciences Center, University of Miami and University of New South Wales IRBs approved reliance agreements based on the UCSF IRB approval.

## Results

Between June 2013 and May 2016, 88 (7.3%) women were identified with HIV infection among the 1198 who completed a baseline CIPI study visit. Their median age was 32 years (IQR 28, 35); 70.45% (n = 62) women reported being HIV infected at study entry, and 49 of these did not elect to be re-tested for HIV by the study. Twenty-six women who were identified as HIV positive on record linkage had self-reported either being HIV negative (N = 23) or never tested (N = 3), most (n = 18) also did not wish to be tested by the study. All women were aware that the study was working with NCHADS to obtain HIV results from testing conducted external to the CIPI Study. HIV-positive women were identified in all ten provinces participating in the CIPI study, but almost half (n = 41) were from two provinces: Phnom Penh (25%; n = 22) and Battambang (21.6%; n = 19). Over one-third (35%) reported never having attended school. Table 1 shows participant characteristics and risk exposures. Women were equally divided (50%) in reporting their primary work setting as either an entertainment venue or as freelance/brothel based FESW. A majority (70%) were members of SMARTgirl, and 15% of members had attended a SMARTgirl club in the past month. All women (99%) reported multiple sex partners in the past 3 months. Unprotected sex in the past 3 months was reported by 38% of women. Twenty-eight women (32%) tested positive for PSA, indicating unprotected sex in the past 48 h. ATS use was prevalent by both self-report (55%) and urine screening (42%; indicating use in the past 48–72 h). We found 81% agreement between self-reported ATS use and ATS urine screening results. On the ASSIST, 51% scored moderate to high-risk for an ATS use disorder, and 51% were classified as being at similarly elevated risk for an alcohol use disorder. There were 27 (31%) participants that scored moderate or high-risk for clinically significant psychological distress on the K-10.

**Table 1:** Characteristics of HIV-positive FESW enrolled in the CIPI Study (N = 88)

Characteristic or exposure	N/median	(%)/IQR
Age (years)		
<20	1	(1)
20–29	27	(31)
30–39	55	(63)
≥40	5	(6)
SMARTgirl member (yes)	62	(70)
Length of time participant has been SMARTgirl member (months) (N = 62)		
<6	25	(40)
6–24	14	(23)
>24	23	(37)
SMARTgirl Club visit frequency in the past year (N = 58^a^)		
≥1 time/month	9	(15)
<1 time/month	35	(60)
Never	14	(24)
Education (total years of school)		
None	31	(35)
Primary (1–6)	44	(50)
Secondary (7+)	13	(15)
Age of first sexual intercourse [median (IQR)]	18	(16–19)
Time since HIV diagnosis at baseline		
0, new infection^b^	29	(33)
1–12 month	18	(20)
>1–5 year	19	(22)
>5 year	22	(25)
Work setting (location worked most days in past month)		
Entertainment venue	44	(50)
Brothel/Freelance	44	(50)
Currently work for manager or boss (yes)	42	(48)
Income last month ($US)^c^		
<100	32	(36)
100–250	36	(41)
>250	20	(23)
Experienced food insecurity (last 3 months)		
Never	54	(61)
Rarely	16	(18)
Sometimes	8	(9)
Often	9	(10)
Experienced any physical or emotional violence (past 3 months)	47	(53)
Number of sexual of partners (last 3 months)		
<5	20	(23)
6–15	27	(31)
16–40	19	(22)
41–150	14	(16)
>150	8	(9)
Number of sexual partners [last 3 months; median (IQR)]	15	(6, 42.5)
Self-reported any unprotected sex (last 3 months)	33	(38)
PSA positive test	28	(32)
Self-reported use of ATS (past 3 months)	48	(55)
Tested for positive for ATS use at study visit	37	(42)
ATS ASSIST score		
<4	37	(42)
4–26	42	(48)
27+	9	(10)
Alcohol ASSIST score		
<11	37	(42)
11–26	33	(38)
27+	18	(20)
K10 Psychological distress (depression and anxiety)		
Low or No risk	37	(43)
Mild risk	23	(26)
Moderate risk	13	(15)
Severe risk	14	(16)
Self-reported symptoms of sexually transmitted infection (last 3 months)	19	(22)

^a^Restricted to the n = 58 SG members with non-missing data

^b^Time since diagnosis was determined by NCHADs records or as the date of testing from CIPI baseline for newly identified infections

^c^Average monthly household income in Cambodia in 2012 was US$56.53; in 2013, US$71.5; in 2014, US$89.98, and in 2015, US$91.14 (https://www.ceicdata.com/en/indicator/cambodia/annual-household-income-per-capita)

**Fig. 1:**
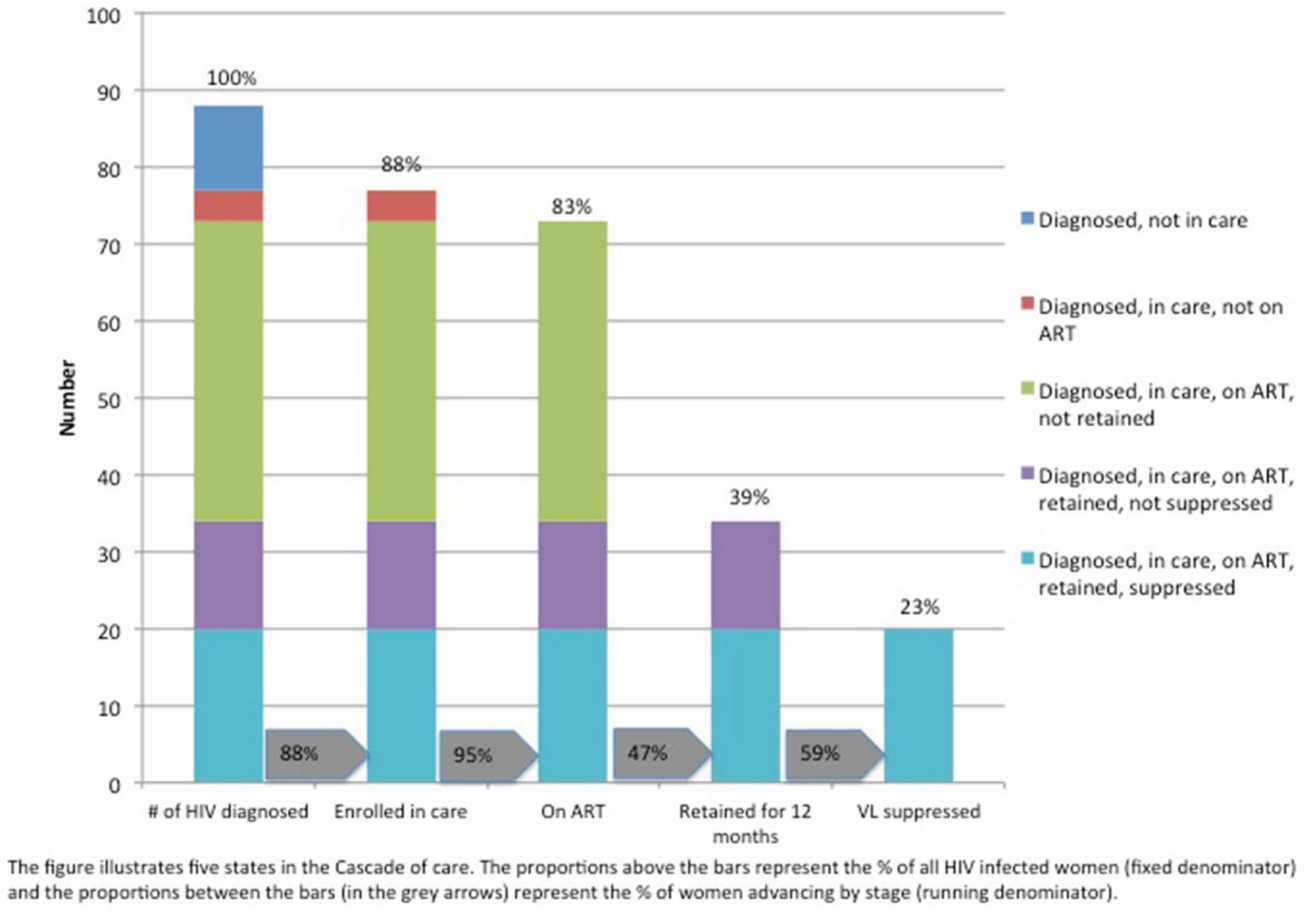
HIV cascade of care among FESW enrolled in the CIPI Study

Figure 1 shows the HIV CoC. Of the 88 HIV-positive women, 88% (n = 77) were enrolled in care, 83% (n = 73) had initiated ART, 39% (n = 34) were retained in ART care at 12 months, and 23% (n = 20) were virally suppressed. The figure shows the proportions that advanced at each stage, and describes the “states” at each stage of the continuum. Using WHO guidelines [10], with a running denominator, we show that 59% of women who had been in care for 12 months were virally suppressed. Records from NCHADS showed that all women classified as virally suppressed (<1000 copies/mL), had undetectable viremia (<50 copies/mL).

Table 2 shows factors associated with 12-month retention in care after initiating ART. In multivariable analyses (Table 2), SMARTgirl membership was independently associated with fourfold greater odds of retention in care (Adjusted Odds Ratio (AOR) = 4.16, 95% CI 1.38, 12.56) compared to women who did not report membership. Women at high risk for an ATS use disorder based on ASSIST scores had 91% lower odds of 12-month retention (AOR = 0.09, 95% CI 0.01, 0.72). Women who reported any STI symptoms in the past 3 months had an elevated but not statistically significant adjusted odds of being retained in care, compared to those who did not report symptoms. Factors independently associated with viral suppression included age [per year increase; AOR 1.17 (95% CI 1.02, 1.34)]; number of sex partners in the past 3 months [per partner increase; AOR 1.01 (95% CI 1.00, 1.02)]; SMARTgirl membership [AOR 8.43; (95% CI 2.44, 29.09)]; self-reporting a sexually transmitted infection in the past 3 months [AOD 16.86 (95% CI 3.02, 94.09)], and scores indicating higher psychological distress on the Kessler (K10) scale (compared to low risk): AOR for Moderate Risk: 14.41 (95% CI 1.81–114.72); AOR for Severe Risk: 7.63 (95% CI 1.13, 51.45).

**Table 2:** Bivariate and multivariable results showing correlates of 12-month retention in HIV care among HIV-positive FESW enrolled in the CIPI study

Characteristic or risk exposure	12 Month retention in HIV Care (n = 34)					
	Bivariate			Multivariable		
	OR	95% CI	*p* value	AOR	95% CI	*p* value
Age (years)						
<20						
20–29	0.88	0.12, 6.29	0.90			
30–39	1.00	0.15, 6.55	1.00			
≥40	1.00	(ref^a^)				
SMARTgirl member (yes)						
Yes	*2.75*	*0.96, 7.82*	*0.06*	*4.16*	*1.38, 12.56*	*0.01*
Length of time participant has been SMARTgirl member (months)						
<6	1.00	(ref)				
6–24	3.83	0.95, 15.36	0.06			
>24	1.95	0.60, 6.35	0.27			
SMARTgirl Club visit frequency in the past year						
≥ 1/month	1.00	(ref)				
<1/month	0.49	0.12, 2.00	0.32			
Never	0.28	0.06, 1.28	0.10			
Age of first sexual intercourse (years)	0.89	0.76, 1.03	0.13	0.84	0.70, 1.02	0.08
Time since HIV diagnosis (months)	1.00	0.99, 1.01	0.67	1.00	0.99, 1.01	0.52
Work setting: Brothel/Freelance based (vs. Entertainment venue)	1.21	0.51, 2.88	0.66			
Currently work for manager or boss	1.71	0.71, 4.08	0.23			
Income last month ($US)						
<100	1.00	(ref)				
100–250	0.64	0.24, 1.73	0.38			
>250	0.86	0.27, 2.68	0.79			
Food insecurity (last 3 months)						
Never	1.00	(ref)				
Rarely	1.32	0.42, 4.13	0.63			
Sometimes	1.02	0.22, 4.77	0.98			
Often	1.36	0.33, 5.71	0.67			
Experienced emotional or physical violence in the last 3 months	1.18	0.49, 2.80	0.71			
Number of sexual partners (self-reported, last 3 months)	1.00	1.00, 1.01	0.37			
Any unprotected sex (self-reported, last 3 months)	0.70	0.28, 1.72	0.43			
PSA positive test	0.66	0.26, 1.71	0.40			
Use of ATS (self-reported, past 3 months) (yes vs. no)	0.50	0.21, 1.21	0.12			
Tested for positive for ATS use at study visit (vs. negative)	0.52	0.21, 1.27	0.15			
ATS assist score						
<4	1.00	(ref)		1	(ref)	
4–26	0.89	0.36, 2.20	0.81	1.20	0.42, 3.38	0.72
27+	0.16	0.02, 1.45	0.11	*0.09*	*0.01, 0.72*	*0.02*
Alcohol assist score						
<11	1.00	(ref)				
11–26	0.51	0.19, 1.38	0.18			
27+	0.75	0.24, 2.37	0.62			
K10 Psychological distress						
Low or no risk	1.00	(ref)				
Mild risk	0.58	0.18, 1.83	0.35			
Moderate risk	2.63	0.71, 9.72	0.15			
Severe risk	0.91	0.25, 3.30	0.89			
STI symptoms (self-reported, last 3 mo)	2.08	0.74, 5.86	0.16	3.00	0.83, 10.81	0.09

Italic values indicate exposures found to be independently and significantly associated with 12-month retention in care

^a^ref = Referent group

## Discussion

This is the first study to characterize the HIV CoC in a group of Cambodian FESW. Although a high proportion of women were successfully linked and enrolled into HIV care, there were precipitous declines in the proportion who remained in care after initiating ART and achieving viral suppression: 43% of women who initiated ART were lost to follow up. These results are in stark contrast to estimates for the general Cambodian population, where 95% of those diagnosed and on ART are retained in care at 12 months [4]. Only one in four (23%) of the HIV-positive women in this study were virally suppressed. The proportion of women on ART who were virally suppressed (59%), however is not dissimilar to estimates in the general Cambodian population (64%) [4]. In contrast to other studies which include female sex workers, our results show that a high proportion were engaged in care and initiated treatment: 94% of those who enrolled in care initiated ART, compared to 28% in a recently published study of CoC outcomes in a South African FSW cohort [33]. Mountain et al. [17] show similar low rates of ART initiation in a meta-analysis of studies of FSW, with a pooled estimate of 39% ART initiation among ART eligible FSW in low and middle income countries. The proportion of virally suppressed participants in our study (59% of women retained) was similar (57%) to the pooled estimate in that study [17]. There are several potential reasons that FESW in Cambodia have poorer retention in care compared to the general population in Cambodia. Women working in the entertainment industry are very mobile, often moving between provinces, which likely impacts retention. Sex work is highly stigmatized in addition to being illegal in Cambodia, and some women may not feel that they can access care that is non-judgmental or responsive to their needs. That viral suppression in women in our sample who are engaged in care is comparable to that seen general population is encouraging. Expanded efforts targeting Cambodian FESW are clearly needed to optimize CoC indicators, especially the goal of successful engagement in care, which would contribute to reductions in HIV morbidity and mortality as well as decreased risk of onward HIV transmission in this high priority population. In Phnom Penh, there are currently two HIV clinics that serve key high-risk groups including FESW, and provide tailored services to women like space for their children during clinic visits. Expanding services that facilitate treatment engagement by FESW could will result in a higher proportion of women who achieve successful viral suppression.

Results also underscore the potential benefits of the SMARTgirl program for facilitating retention in care. SMARTgirl membership was associated with more than fourfold greater odds of 12-month retention after initiating ART, which highlights the potential benefits of continued implementation of SMARTgirl to optimize HIV treatment as prevention in this population. It was also associated with over eightfold higher odds of viral suppression. The SMARTgirl program, aimed at FESW, is designed to engage women in community HIV prevention efforts, principally by encouraging HIV testing and providing referrals to care for STIs and HIV infections. The SMARTgirl club and membership therein may be providing a larger platform for engagement and social mobilization [34] and which could be operationalizing broadly as a empowerment opportunity [35]. The broad reach of SMARTgirl could potentially be further leveraged as a platform to reach higher risk women, as has been done with the CIPI project [26], to implement multicomponent HIV prevention and care interventions.

Approximately half of participants screened positive for an ATS use disorder and/or an alcohol use disorder, and one-third screened with clinically significant psychological distress. Consistent with findings from the U.S. [19], those at highest risk for an ATS use disorder had 91% lower odds of 12-month retention after initiating ART. Expanded intervention approaches targeting ATS use, including conditional cash transfer with a cognitive-behavioral aftercare group are being tested as part of the CIPI trial [26, 27]. If this intervention proves effective in reducing ATS use, it could be more widely implemented using the SMARTgirl platform to address gaps in the HIV CoC among FESW. The high prevalence of psychiatric distress is noteworthy. Our findings are consistent with another recent study in a sample of FESW from Phnom Penh and Siem Reap, wherein almost half (43.2%) screened positive for psychological distress [36]. In our analyses of correlates of viral suppression, women with increased risk of psychiatric distress had higher odds of viral suppression. Further research should be done to examine this association, including the directionality and temporality of ART uptake and psychological well-being in FESW.

Multiple overlapping circumstances contribute to high HIV risk among Cambodian FESW, including individual sexual and drug risk exposures, low education and high poverty levels, and the negative impacts of public policy and policing [3]. In particular, violence serves as an important trigger for ATS use, and it is independently associated with unprotected sex among Cambodian FESW [6, 8, 9]. Central to many women’s employment in entertainment venues is the requirement for highly sociable interactions with male customers and, for some women, this extends to transactional sex as an opportunity for income generation. ATS are used by many women to stay awake and work longer and see more clients, which also contributes to HIV risk [2, 37]. Expanded access to ART now joins the backbone HIV prevention platform of HIV testing, condom promotion and diagnosis, and treatment of STI as Cambodia aims for HIV elimination.

Our study has several limitations. First, the sample size is small, and results may not reflect the HIV CoC among all Cambodian FESW living with HIV. For some women we relied on self-report HIV status; however only women who reported a previous positive test were not tested by the study. Some (n = 26) women were diagnosed with HIV by the study, and although we obtained data after a year of diagnosis, including this recently diagnosed group could have led to conservative estimates of 12-month retention following ART initiation and viral suppression during this period. Viral suppression is defined in Cambodia as viral loads <1000 copies/mL, and this definition could potentially include women who had previously been virally suppressed, but who subsequently experienced breakthrough viremia and were no longer suppressed. The low numbers of viral suppression outcomes also impact risk estimates, and measures of excess risk found in multivariable analyses predicting this outcome should be interpreted with caution due to wide confidence intervals. This is an observational study, and the association seen with SMARTGirl membership should be interpreted with caution as this could reflect population characteristics rather than the effects of the program.

Despite these limitations, this study is the first to characterize the HIV CoC in Cambodian FESW, articulate the benefits of targeting this population for optimizing retention in HIV care, and identify ATS use as a risk factor for sub-optimal retention following ART initiation. These findings provide important information for sustaining and strengthening combination HIV prevention with this high priority population in order to eliminate HIV transmission in Cambodia by 2020.

There is increasing awareness that to meet the ambitious UNAIDS 90-90-90 HIV treatment targets and to get to zero new infections, efforts must be made to extend services and retain in care those who are most disenfranchised and at the highest risk for HIV disease progression and onward HIV transmission [38]. Improvements in the CoC states will optimize health outcomes and reduce HIV transmission risk [39]. Targeting FESW who use ATS will optimize the benefits of ART treatment. Leveraging existing programmatic platforms such as SMARTgirl, will enhance coverage to HIV-positive FESW, and potentially reduce attrition at all stages of the CoC.
